# A review of the genus *Ogdoconta* Butler (Lepidoptera, Noctuidae, Condicinae, Condicini) from North America north of Mexico with descriptions of three new species

**DOI:** 10.3897/zookeys.264.4060

**Published:** 2013-02-06

**Authors:** Eric H. Metzler, Edward C. Knudson, Robert W. Poole, J. Donald Lafontaine, Michael G. Pogue

**Affiliations:** 1 Michigan State University Adjunct Curator of Lepidoptera; Research Collaborator, U.S.N.M. Natural His-tory Museum; P.O. Box 45, Alamogordo, NM 88311-0045 USA; 2 Texas Lepidoptera Survey, 8517 Burkhart Rd., Houston, TX 77055-7517 USA; 3 Research Associate, Department of Entomology, Smithsonian Institution, Washington, DC 20013-7012 USA; 4 Canadian National Collection of Insects, Arachnids and Nematodes, Agriculture and Agri-Food Canada, K. W. Neatby Building, 960 Carling Ave., Ottawa, ON K1A 0C6 Canada; 5 Systematic Entomology Laboratory, PSI Agricultural Research Service, US Department of Agriculture, c/o Smithsonian Institution, P.O. Box 37012, NMNH MRC-168, Washington, DC 20013-7012 USA

**Keywords:** Biological diversity, Condicinae, Condicini, Arizona, New Mexico, Texas, *Ogdoconta*, Carlsbad Caverns National Park, Louisiana, Mississippi, Florida

## Abstract

The species of the genus *Ogdoconta* Butler, 1891 (Lepidoptera, Noctuidae, Condicinae, Condicini) from North America north of Mexico are reviewed, and a description of the genus is given. *Ogdoconta satana* Metzler, Knudson & Poole, **sp. n.**, is described from New Mexico and Texas, *Ogdoconta rufipenna* Metzler, Knudson & Poole, **sp. n.**, is described from Arizona, and *Ogdoconta fergusoni* Metzler & Lafontaine, **sp. n.**, is described from Florida, Mississippi, and Louisiana. A key to the species of *Ogdoconta* of North America north of Mexico is provided. Adult moths and male and female genitalia of *Ogdoconta satana*, *Ogdoconta rufipenna*, *Ogdoconta fergusoni*, *Ogdoconta cinereola* (Guenée, 1852), *Ogdoconta moreno* Barnes, 1907, *Ogdoconta sexta* Barnes & McDunnough, 1913, *Ogdoconta altura* Barnes, 1904, and *Ogdoconta tacna* (Barnes, 1904) are illustrated.

## Introduction

Poole (1989) listed 13 species of *Ogdoconta* Butler, 1891 (Noctuidae, Condicinae, Condicini), all from the New World. In 2010, Lafontaine and Schmidt listed five described species of *Ogdoconta* from North America north of Mexico: *Ogdoconta cinereola* (Guenée, 1852), described from New York, USA; *Ogdoconta moreno* Barnes, 1907, described from Pima Co., Arizona, USA; *Ogdoconta sexta* Barnes & McDunnough, 1913, described from Brownsville, Cameron Co., Texas, USA; *Ogdoconta altura* Barnes, 1904, described from Kerrville, Kerr Co., Texas, USA; *Ogdoconta tacna* (Barnes, 1904), described from Kerrville, Kerr Co., Texas, USA; and one undescribed species, *O*. sp. not *Ogdoconta lilacina* (Druce, 1890), recorded from southern Arizona, USA. A name, *Ogdoconta rufipenna*, is provided for the undescribed species listed by Lafontaine and Schmidt (2010). Our investigations show that two additional undescribed species of *Ogdoconta* occur in the United States. *Ogdoconta satana* occurs in New Mexico and Texas, and *Ogdoconta fergusoni* occurs in Florida, Mississippi, and Louisiana.

## Material and methods

Genitalia were examined following procedures outlined in Clarke (1941), Hardwick (1950), Lafontaine (2004), and Pogue (2002). Abdomens were removed, soaked in KOH, dissected, and slide mounted.

Wing pattern terminology is from Mikkola et al. (2009). Morphological structure terminology is from Richards (1933) and Scoble (1995). Genital structure terminology is from Lafontaine (2004), Klots (1970), and Mikkola et al. (2009). Forewing lengths, taken from wing base to apex, excluding fringe, were measured to the nearest one half mm using a stereo-microscope with a reticle. All specimens from New Mexico were collected as part of a ten-year faunal study of Lepidoptera of Carlsbad Caverns National Park.

Specimens of Lepidoptera cited in this study are deposited in the following collections (collection abbreviations where available from Evenhuis (1993)):

**BMNH** Natural History Museum, London, England (statutorily, British Museum of Natural History)

**CDF** Clifford D. Ferris, Laramie, Wyoming

**CNC** Canadian National Collection of Insects, Arachnids and Nematodes, Ottawa, Ontario

**CUIC** Cornell University Insect Collection, Ithaca, New York

**EHM** Eric H. Metzler, Alamogordo, New Mexico, for subsequent transfer to MSUC

**JBW** J. Bruce Walsh, Tucson, Arizona

**LACM** Natural History Museum of Los Angeles County, Los Angeles, California

**MEM** Mississippi Entomological Museum, Mississippi State University, Mississippi State, Mississippi

**MSUC** Albert J. Cook Arthropod Research Collection, Department of Entomology, Michigan State University, East Lansing, Michigan

**TLSRC** Texas Lepidoptera Survey Research Collection, Edward C. Knudson, Houston, Texas

**UNMC** Museum of Southwestern Biology, University of New Mexico, Albuquerque, New Mexico

**USNM** National Museum of Natural History, Smithsonian Institution, Washington, DC

**VAB** Vernon A. Brou, Jr., Abita Springs, Louisiana

## Results

### Key to the species of *Ogdoconta* in North America north of Mexico

**Table:** 

1	Forewing solidly suffused with ash black, reniform and orbicular spots pale filled or obscure; postmedial line deeply zigzagged and without a pale area along distal margin	*Ogdoconta satana*
–	Forewing not solidly suffused with ash black, but if mainly black, then with pale whitish-gray band by subterminal line; reniform and orbicular spots outlined in white in most species, but represented by diffuse pale patches in two species, postmedial line straight or sinuate, with pale band along distal side in most species	2
2	Forewing color solidly red brown with purple tint (burgundy)	*Ogdoconta rufipenna*
–	Forewing color not burgundy	3
3	Forewing pale or dark clay-colored gray, medial area, particularly near postmedial line, darker than subterminal area; reniform spot distinctly lighter than medial area around it	*Ogdoconta moreno*
–	Forewing not pale or dark clay-colored gray, reniform spot not distinctly lighter than medial area around it	4
4	Forewing with postmedial line slightly sinuous, at 90° angle to posterior margin, curved outward to distal end of reniform, then broadly curved around reniform, turning basad towards costa	5
–	Forewing with postmedial line nearly straight from posterior margin to just before costa, not forming 90° angle at posterior margin	7
5	Larger (forewing length 11.0–13.5 mm)	6
–	Smaller (forewing length 9.0–10.5 mm)	*Ogdoconta fergusoni*
6	Forewing with subterminal area lighter than medial and terminal areas; postmedial area usually shaded with pink (specimens from southern Arizona with less pink); hind wing of both sexes solidly dark brown	*Ogdoconta cinereola*
–	Forewing with subterminal area barely lighter than medial and terminal areas; hind wing of male white; hind wing of female pale at base, darker towards outer margin	*Ogdoconta tacna*
7	Costa of forewing with a small streak of white scales extending from just before apex to just below inward angle of postmedial line; valve of male genitalia with cucullar region ovate, capitate; ostium of female genitalia flaring, opening larger than caudal part of ductus bursae	*Ogdoconta sexta*
–	Costa of forewing without a small streak of white scales in apical area; valve of male genitalia with cucullar region more linear, not ovate; ostium of female genitalia not flaring, opening not significantly larger than caudal part of ductus bursae	*Ogdoconta altura*

#### Ogdoconta

Butler

http://species-id.net/wiki/Ogdoconta

Ogdoconta Butler, 1891: 462.

##### Type species.

*Placodes cinereola* Guenée, by original designation.

##### Diagnosis.

*Ogdoconta* is a moderately-sized (approximately 15 species) New World genus with its largest concentration of species in the southwestern United States, Mexico, and Central America. The North American species of *Ogdoconta* are small to moderate in size (forewing length 9.5–16.0 mm), and their appearance is not particularly distinctive, because there are no external characters that uniquely characterize this genus. There are, however, characters in both the male and female genitalia unique to *Ogdoconta*. The most distinctive feature is the divided valve in the male genitalia. The saccular and cucullar regions are separate, although joined at the base. In the female genitalia the junction of the ductus bursae, corpus bursae, and appendix bursae are proximate.

##### Description.

*Head*: Male and female antennae scaled above, naked below; occiput with dentate scales projecting slightly between antennal bases; a rough, poorly-developed line of scales just below antennal bases; front smoothly scaled, without frontal process, raised ring, or other modifications; eye normal, rounded, without hair; ocelli present; no lashes from either base of antenna or rear margin of eye; palpus unmodified, upturned, reaching about midpoint of front; third segment about one-third length of second; both second and third palpal segments smoothly scaled, first segment with a ventral tuft of scales; haustellum normal. *Thorax*: Wing venation normal for trifid noctuids; dorsum of thorax covered with elongate scales. Frenulum in female occasionally with a single bristle. Prothoracic leg: femur closely covered with flat scales except for a few rough scales projecting along ventral margin; tibia about one-half as long as femur, smoothly scaled, except for a few rough scales along outer margins; tibial claws and spines absent; first tarsal segment almost as long as tibia; first three tarsal segments scaled both dorsally and ventrally, fourth and fifth tarsal segments may have reduced scaling, fifth tarsal segment mostly naked ventrally; tarsal segments with three rows of spines ventrally. Mesothoracic and metathoracic legs: femur about four times longer than wide, smoothly scaled, except for fringe of long hairs along ventral margin; tibia scaling rougher than on femur; usual one mesotibial and two metatibial pairs of spurs; tarsal segments as in prothoracic leg. External tympanal region with alula strong; tympanic membrane circular, not elongate. *Male abdomen*: Weak tufts of scales present or absent on dorsum of segments one and two; sternite 1+2 without basal hair pencils or Stobe’s glands; sclerotized margin of eighth sternite appears U-shaped; eighth sternite with two separate strong lines of hairs, not clearly separated into two hair pencils; sclerotized region of eighth tergite Y-shaped. *Male genitalia*: Uncus straight, narrow, often hairy; scaphium variously sclerotized; subscaphium variously sclerotized; anal tube variously sclerotized; tegumen simple, with or without slight projecting lobe near articulation with vinculum; tegumen hairy; vinculum and tegumen articulating directly, neither extensions, processes, nor separate pleurites present; juxta variously triangular, drawn out posteriorly; valve with saccular and cucullar regions separated giving valve a bifid appearance; cucullar region rectangular, ovate, or elongate; mesial surface of distal part of cucullar region simple and densely hairy, projections from costal margin present or absent; type species with a clasper located near junction of cucullar and saccular parts of valve, clasper-like structure present or absent in other species; saccular part of valve long, shorter than cucullar part, narrow, mesially densely covered with hairs; shaft of aedeagus narrow, with slight apical process variable; vesica long, from one half loop to two full loops, with or without diverticula or spines. *Female genitalia*: Ovipositor lobes unmodified, setae present; anterior apophyses and posterior apophyses, short, approximately equal in length; ostium in membrane between seventh and eighth abdominal segments, closer to eighth segment, with varying amounts of sclerotization; ductus bursae generally narrow, sclerotized or membranous, of variable length; appendix bursae wide, membranous; corpus bursae globular, slightly narrowed at junction with ductus bursae and appendix bursae; signa present or absent.

##### Discussion.

In North America north of Mexico, the species of *Ogdoconta* are placed in three groups. The first group (here called the *cinereola* species group) contains five species; *Ogdoconta cinereola*, *Ogdoconta moreno*, *Ogdoconta sexta*, *Ogdoconta altura*, and *Ogdoconta satana*. This group has a simple divided valve without projections on the outer margin. The vesica of the aedeagus is elongate with a single large loop. In the female genitalia the ostium is sclerotized, partially sclerotized or membranous. The ductus bursae is variable in length, narrow or funnel shaped, straight or bent, and membranous or partially sclerotized. A single junction joins the ductus bursae, corpus bursae, and appendix bursae. The corpus bursae is globular, usually with a single concave signum. The appendix bursae is large and fully or partially coiled. The junction between the appendix bursae and ductus seminalis may not be distinct.

*Ogdoconta cinereola* differs from all other species in the group in that *Ogdoconta cinereola* has a small clasper at the juncture of the cucullar and saccular regions of the valve. *Ogdoconta altura*, *Ogdoconta sexta*, *Ogdoconta moreno*, and *Ogdoconta satana* have no clasper. The distribution of *Ogdoconta cinereola* generally falls outside that of the other four species in the *cinereola* group.

The second group contains two species *Ogdoconta tacna* and *Ogdoconta fergusoni*. The outer margin of the cucullar part of the valve is less curved than the *cinereola* group, and the outer margin of the cucullar part has one or more projections. The vesica of the aedeagus has two rows of short, stubby spines. The female genitalia of the two species in the *tacna* group are dissimilar from each other, and they are dissimilar from the other species groups.

The third group contains the single species *Ogdoconta rufipenna* that is characterized by the cucullar part of the valve, which is noticeably more narrow than the other species groups. The outer margin of the cucullar part of the valve has a conspicuous thumb-like projection. In the female genitalia the ostium is strongly sclerotized and the scler-otization continues down the ductus bursae. The ductus bursae, supported by the terminus of the sclerotization, is bent.

### *cinereola* species group

#### Ogdoconta cinereola

(Guenée, 1852)

http://species-id.net/wiki/Ogdoconta_cinereola

**Figures 1–10.**
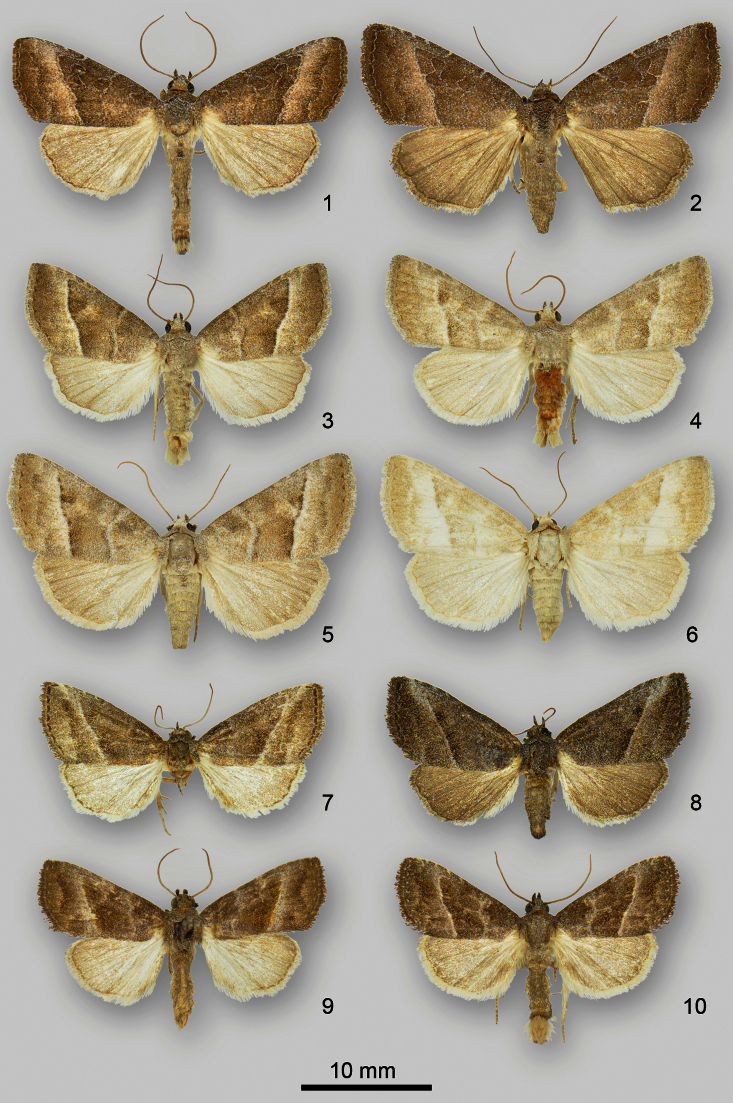
*Ogdoconta* adults. **1** *Ogdoconta cinereola* male **2** *Ogdoconta cinereola* female **3** *Ogdoconta moreno* male **4** *Ogdoconta moreno* male **5** *Ogdoconta moreno* female **6** *Ogdoconta moreno* female **7** *Ogdoconta sexta* male **8** *Ogdoconta sexta* female **9** *Ogdoconta altura* male **10** *Ogdoconta altura* female

Figs 1, 2 , 20, 21 , 36

Placodes cinereola Guenée, 1852: 316, pl. 15, fig. 1.
Miana atomaria Walker, 1865: 675.

##### Type material.

*Placodes cinereola* was based on two syntypes from New York, USA, from the Boisduval and Doubleday collections. A single syntype, labeled "U. S. America, Doubleday, 46–110" with a handwritten label "*Placodes cinereola*", now extant in the BMNH from the Doubleday collection, is labeled and hereby designated as **Lectotype** to ensure the stability of the name. Walker based *Miana atomaria* on three syntypes from the United States that were in the BMNH. The syntypes could not be located in the BMNH and Hampson (1910) does not list any types in his catalogue.

##### Diagnosis.

Forewing is light fuscous brown, and the subterminal region (between the postmedial and subterminal lines) is suffused with a pinkish tinge. Medial and basal areas are minutely speckled with white. Antemedial line is an obscure, scalloped white line. Reniform and orbicular spots are obscure but often discernible by fine white outlines. Claviform spot is absent. Postmedial line is a white, almost straight, oblique line with a slight basally directed bend at CU_2_. Subterminal line is marked primarily as a brown shade terminating the pink suffusion of the subterminal region. Hind wing is suffused with brown. Males and females are similar in appearance, although the female hind wing usually is darker. Forewing length: 9.5–14.5 mm. This appears to be the only species in the genus with a clasper near the junction of the saccular and cucullar regions of the valve.

##### Distribution and biology.

*Ogdoconta cinereola* is the only widely distributed and commonly collected species of *Ogdoconta* in eastern, central, and southwestern North America. It occurs from southern Ontario and Quebec south to southern Florida. At the western edge of its distribution, *Ogdoconta cinereola* occurs from Manitoba southward through the Great Plains of Nebraska and Iowa, south throughout most of Texas, and westward through southern New Mexico (Eddy County) to southeastern Arizona (Santa Cruz County). The distribution extends south to the state of Coahuila in northern Mexico. Reports of this species from British Columbia are based on a mislabeled specimen; several other species from the same collection, now in the CNC, are also mislabeled as to locality with the same “Vancouver, B.C.” label.

The larva of *Ogdoconta cinereola* was described by Coquillett (1880), Hampson (1910), Crumb (1956), and Wagner et al. (2011). Wagner et al. (2011) provided pictures of the larva. Recorded larval hosts include five plant families; Amaranthaceae, Asteraceae, especially *Ambrosia* spp. (ragweeds), Fabaceae, Labiatae, and Poaceae (Ashmead 1886, Crumb 1956, Tietz 1972, Robinson et al. 2002, Heppner 2003, Wagner et al. 2011, Robinson et al. 2012).

**Figures 11–19.**
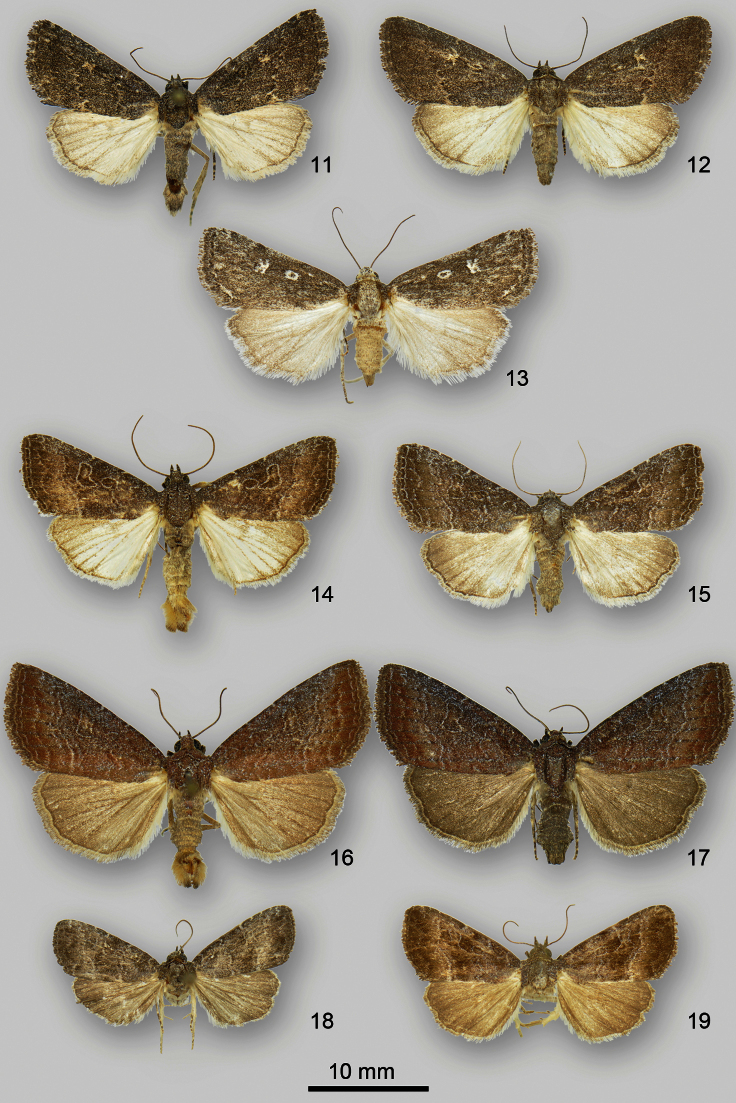
*Ogdoconta* and *Fotella notalis* adults. **11** *Ogdoconta satana* male **12** *Ogdoconta satana* female paratype **13** *Fotella notalis* dark female **14 ***Ogdoconta tacna* male **15** *Ogdoconta tacna* female **16** *Ogdoconta rufipenna* male holotype **17** *Ogdoconta rufipenna* female paratype **18** *Ogdoconta fergusoni* male holotype **19** *Ogdoconta fergusoni* female paratype.

##### Remarks.

This moth is easy to identify because of the pink in the subterminal area of the forewing. The adults are generally common and occur from May to September in the north, to as early as April and as late as October, in Texas and Florida. The saturation of pink in the postmedial area is reduced in specimens from southern Arizona. The pink postmedial area in some individuals is wider. Varying portions of the basal area of some specimens is replaced with pink.

#### Ogdoconta moreno

Barnes, 1907

http://species-id.net/wiki/Ogdoconta_moreno

Figs 3–6 , 22, 23 , 37

Ogdoconta moreno Barnes 1907: 96.

##### Type material.

*Ogdoconta moreno* is based on an unspecified number of syntypes. There are currently two syntypes in the USNM bearing Barnes’ syntype labels, a male and a female. The male syntype bearing the locality “Baboquivera [sic] Mts., Pima Co., Ariz.” is labeled and hereby designated as **Lectotype** to ensure the stability of the name.

##### Diagnosis.

Adults of *Ogdoconta moreno* vary from brown to gray. This species is not likely to be confused with any other species of *Ogdoconta* in North America. Both the reniform and orbicular spots of the forewing are represented by contrasting light patches devoid of any defining lines or spots. Orbicular spot touches the antemedial line. Antemedial line is angled with the outward apex occurring just below the orbicular spot. Inner side of the antemedial line is a light band followed by a darker brown line. Postmedial line is an almost straight, light line, followed by a light tan or gray region of the subterminal area, which gradually becomes darker in the subterminal area. Medial and terminal areas of the forewings of individual specimens, of both sexes, range from pale tan to dark smoky gray. Hind wing of both the male and female is whitish, suffused with dull gray brown, more heavily in the female than the male. Forewing length: 10.0–14.0 mm.

Cucullar part of the valve is ovate, elongate, and the outer margin is unmodified. Vesica loop varies from 180° to 360°. Vesica has a prominent curved diverticulum. Female genitalia are almost entirely membranous. Corpus bursae is elongate with a conspicuous signum that is concave and imbedded with minute denticles.

**Figures 20–27.**
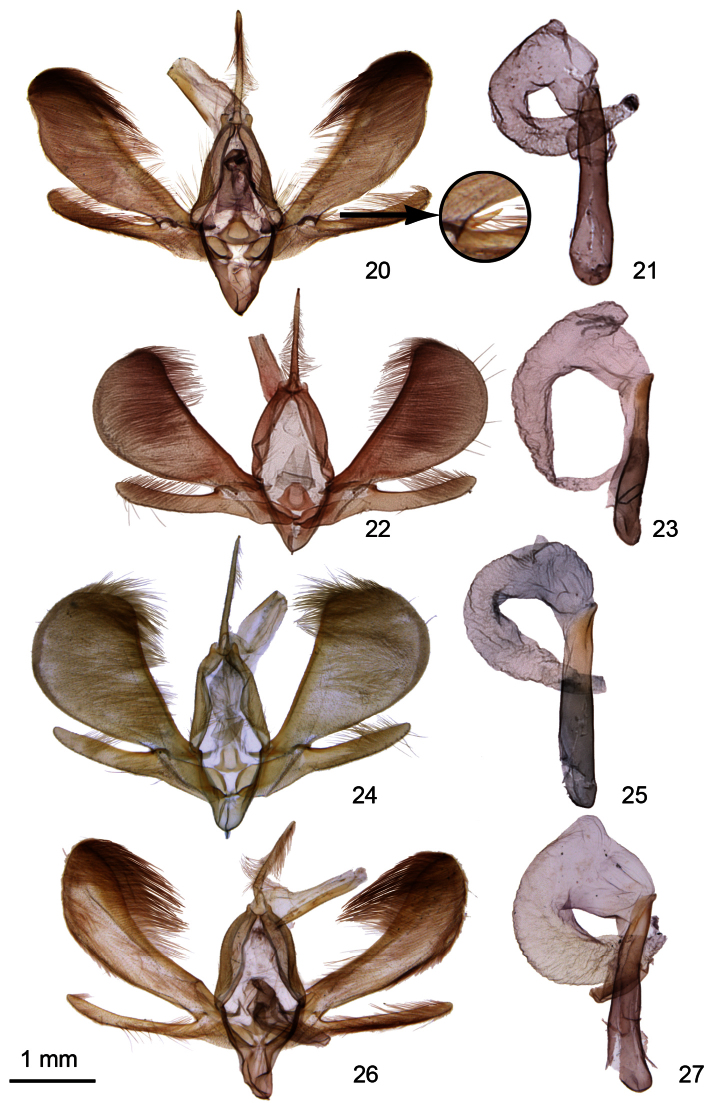
*Ogdoconta* male genitalia. **20** *Ogdoconta cinereola* with insertedenlargement showing detail of *Ogdoconta cinereola* clasper **21** *Ogdoconta cinereola* aedeagus **22** *Ogdoconta moreno*. **23** *Ogdoconta moreno* aedeagus **24** *Ogdoconta sexta* **25** *Ogdoconta sexta* aedeagus **26** *Ogdoconta altura* **27** *Ogdoconta altura* aedeagus.

##### Distribution and biology.

This species is known only from southern Arizona, although its distribution likely extends into Mexico. The larva and its food plants are unknown. Adults were collected in July, August, and September.

#### Ogdoconta sexta

Barnes & McDunnough, 1913

http://species-id.net/wiki/Ogdoconta_sexta

Figs 7, 8 , 24, 25 , 38

Ogdoconta sexta Barnes and McDunnough 1913: 117, pl. 5 fig. 6.

##### Type material.

*Ogdoconta sexta* is based on an unspecified number of syntypes. There are currently two syntypes in the USNM labeled “Type ♂” and “Type ♀” respectively. The male syntype bearing the locality “Brownsville, Texas” is labeled and hereby designated as **Lectotype** to ensure the stability of the name.

##### Diagnosis.

The best superficial character to separate *Ogdoconta sexta* from *Ogdoconta altura* is found near the apex of the forewing. *Ogdoconta sexta* has a small streak of white scales, best seen with low magnification, from just before the apex of the wing to just below the outward angulation of the postmedial line. This feature gives the forewing costa of *Ogdoconta sexta*, just before the apex, a frosted appearance (Figs 7, 8). Otherwise, the region following the postmedial line of *Ogdoconta sexta* is not lighter than the rest of the subterminal area. Most of the maculation of the forewing is obscure. However the postmedial line is moderately distinct, straight, except for a slight basally directed angulation near the costa, and white or yellow in color. Hind wing is suffused with dull brown; hind wings of some males are not as dark as the hind wings of females. There is no geographical variation. To the naked eye, *Ogdoconta sexta* appears slightly larger than *Ogdoconta altura*; the wing measurements show them to be the same size. Forewing length: 10.5–13.0 mm.

In the male genitalia the apex of the cucullar region of the valve is ovate-capitate in *Ogdoconta sexta*, but narrower and more lineate in *Ogdoconta altura*. In the female genitalia of *Ogdoconta sexta* the ostium is wider than long, but the ostium of *Ogdoconta altura* is longer than wide or the two measurements are approximately equal. The ostium of *Ogdoconta altura* appears larger when compared to the ostium of *Ogdoconta sexta*.

##### Distribution and biology.

*Ogdoconta sexta* is known from Hidalgo and Cameron Counties in the southernmost Texas. Its distribution in Mexico is not known. The larva and its food plants are unknown. Adults were collected from March to June and again in September. *Ogdoconta sexta* is infrequently collected.

##### Remarks.

*Ogdoconta sexta* and *Ogdoconta altura* are difficult to separate superficially. Both species are similar in size, and the overall coloration is brown with a green-gray cast. Males tend to be slightly lighter than females, and in particular the subterminal area of the male can be significantly lighter than the medial area. A few males have significantly weaker brown suffusion in the hind wing than do other males and all females. When held in the light at the correct angle, the wings appear to be shiny.

#### Ogdoconta altura

Barnes, 1904

http://species-id.net/wiki/Ogdoconta_altura

Figs 9, 10 , 26, 27 , 39

Ogdoconta altura Barnes 1904b: 243.

##### Type material.

*Ogdoconta altura* is based on an unspecified number of syntypes. The USNM has three syntypes, 2 females and 1 male with red-bordered type labels that are verifiably part of the original syntype series. The male syntype bearing the locality “Kerrville Texas” is labeled and hereby designated as **Lectotype** to ensure the stability of the name.

##### Diagnosis.

The best character to separate *Ogdoconta altura* from *Ogdoconta sexta* is found near the apex of the forewing. *Ogdoconta altura* is lacking the small smear of white scales near the apex described in the diagnosis of *Ogdoconta sexta*. In *Ogdoconta altura*, the forewing costa proximal to the apex, lacks a frosted appearance (Figs 9, 10). The region following the postmedial line in *Ogdoconta altura* is lighter than the rest of the subterminal area. Forewing length: 9.5–13.0 mm.

The genital differences between *Ogdoconta altura* and *Ogdoconta sexta* are given in the diagnosis of *Ogdoconta sexta*.

##### Distribution and biology.

*Ogdoconta altura* has a wider distribution than *Ogdoconta sexta*. *Ogdoconta altura* was collected in south central and southern Texas as well as in northeastern Mexico. The larva and its food plants are unknown. Adults were collected in April, May, July, August, and September. *Ogdoconta altura* is infrequently collected.

##### Remarks.

*Ogdoconta altura* and *Ogdoconta sexta* are difficult to separate superficially. Both species are similar size, and the overall coloration is brown with a green-gray cast. When held in the light at the correct angle, the wings appear to be shiny. The hind wing of the female is more heavily suffused with brown than the male, although the amount of dark suffusion is variable among males. Among males, some individuals have a smoother and slightly lighter appearance than others. There is no geographic variation.

#### Ogdoconta satana

Metzler, Knudson & Poole sp. n.

urn:lsid:zoobank.org:act:BB9545A9-6716-4AAC-9B52-42E77E1D104B

http://species-id.net/wiki/Ogdoconta_satana

**Figures 28–35.**
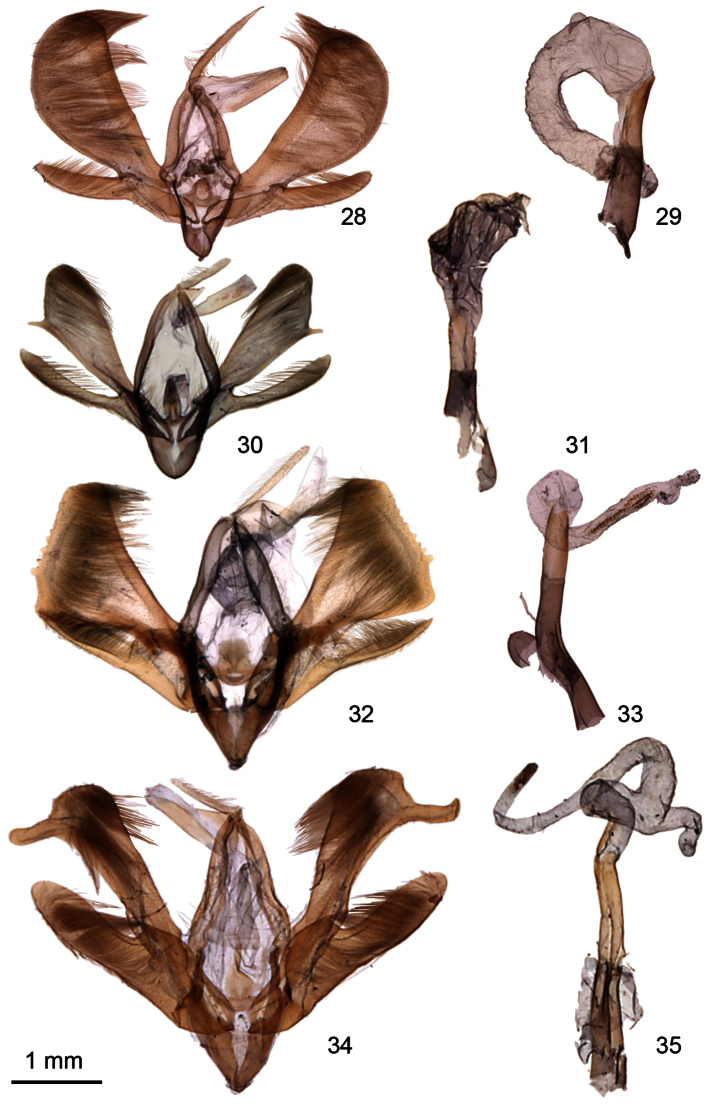
*Ogdoconta* male genitalia. **28** *Ogdoconta satana* **29** *Ogdoconta satana* aedeagus **30** *Ogdoconta fergusoni* **31** *Ogdoconta fergusoni* aedeagus with vesica partially everted **32** *Ogdoconta tacna* **33** *Ogdoconta tacna* aedeagus **34** *Ogdoconta rufipenna* **35** *Ogdoconta rufipenna* aedeagus.

Figs 11, 12 , 28, 29 , 40, 44

##### Type material.

**Holotype**: Adult male, pinned: USA: NM: Eddy Co. Carlsbad Caverns N[ational ]P[ark], arroyo habitat, 104°33.74'W, 32°06.38'N 4,170’, 10 July 2010, CCNP5, Eric H. Metzler, uv tr[a]p, Accss #: CAVE - 02263 (USNM). **Paratypes:** 38 males and 20 females: Same data as holotype with dates 10 July 2010, 9 June 2009, 14 June 2007. USA: NM: Eddy Co. Carlsbad Caverns NP, arroyo habitat, 104°33.569'W, 32°5.976'N 4,100’, 9 June 2009, 6 August 2010 CCNP2, Eric H. Metzler, uv trp, Accss #: CAVE - 02263. USA: NM: Eddy Co. Carlsbad Caverns NP, grassland habitat, 32°06.222'N 4,160’ 104°33.759'W, 29 August 2006, CCNP1, Eric H. Metzler Accss #: CAVE - 02263. TEXAS, Culberson Co., Guadalupe Mts. NP, Williams Ranch, 10,11-VIII-91, leg.E.C.Knudson. TEXAS: Culberson Co. Sierra Diablo WMA, leg. E.C.Knudson, 18-VIII-84. TX: Culberson Co., Guadalupe MtsNP, Ship on Desert, 24-VIII-95, leg.E.Knudson. Texas: Bear Canyon, Guadalupe Mts., Texas, 5700’, 4.IX.69, A. & M.E. Blanchard. TX: El Paso Co., Hueco Tanks SP, 23-IX-95/ ECK. TX: Brewster Co., Big Bend NP, Dugout Wells, 8-IX-10 B/K. Shafter, Presidio County, Texas, 9.IX.69, A. & M.E. Blanchard. Sierra Diablo Wildlife Mgt. Area, 6000’, Culberson Co., Tex., 12–15.VII.71. (USNM, UNMC, MSUC, EHM, TLSRC).

##### Etymology.

The scientific name *satana* comes from the Marvel comic book fictional character Satana, a child of Satan and sinister character, who taught black magic. The name refers to the black (often equated with evil) color of the adult moth. It is treated as a noun in apposition.

##### Diagnosis.

*Ogdoconta satana* is an easily recognized species within the genus *Ogdoconta*. Forewing is completely suffused with dark ash black, and hind wing is contrastingly pale. Most of the maculation is obscure; scalloped black postmedial line is barely visible. Both orbicular and reniform spots are present as small contrasting light spots or are obscure. Orbicular spot is small and round with an ash-black center, and the reniform spot, filled with ash black, is obscure towards costa and posterior margin. Under low magnification the medial area may appear to be darker. Hind wing is contrastingly pale. There is little or no variation in the appearance of the upperside of *Ogdoconta satana*; underside, in both males and females, can vary from dirty white with scattered dark-fuscous scales to dark fuscous with scattered dirty-white scales. Orbicular and reniform spots on the underside range from prominent to obscure, and the color may be pale gray, yellow, or dirty white, filled with dark gray, or black. Under low magnification, forewings of some specimens may appear to have a dark-brown tint. No other species of *Ogdoconta* in North America shares these characters.

Females of *Ogdoconta satana* are superficially similar to very dark females of *Fotella notalis* Grote, 1882 (Noctuidae, Condicinae, Leuconyctini) (Fig. 13). The two species are sympatric in the northern Chihuahuan Desert, and they are easy to distinguish. The front of *Fotella notalis* has a caldera-like raised ring with a depressed center. Scales between the raised ring and the clypeus are dirty-white, and the antennal scape of *Fotella notalis* is white. The front of *Ogdoconta satana* does not have a raised ring. Scales near the clypeus and the antennal scape of *Ogdoconta satana* are black. Also, *Ogdoconta satana* has a broader forewing than does *Fotella notalis*.

##### Description.

**Adult male** (Fig. 11). *Head*: Ash black, scales spatulate white tipped, vertex scales erect, front scales smooth. Labial palpus erect, smooth ash black, scattered white scales, scales spatulate, basal segment with ventral tuft of scales, second segment apex mesially white. Haustellum coiled between labial palpi. Antenna filiform, sensory setae inconspicuous, each segment dorsally alternating gray and pale, scales appressed, ventrally naked, light brown. *Thorax*: Ash black, scales spatulate white tipped; underside dirty white, scales appressed, scattered long hair-like scales. Legs dorsally ash black, ventrally dirty white, smoothly scaled, sparse long hair-like white scales; tarsomeres ash black, white tipped. Forewing: length 10.0–13.0 mm, mean 11.5 mm, n = 37; dorsal surface ground color ash black with scattered gray scales. Basal line obscure, faintly darker; antemedial line obscure, faintly darker; postmedial line dirty white at posterior margin, pointed basally on veins, obscure over cell, disappearing towards costa; subterminal line obscure; terminal line obscure; orbicular spot a round dirty-white ring filled with ash black; reniform spot a kidney-shaped dirty-white ring filled with ash black, obscured towards costa and posterior margin; costa with four or five small dirty-white spots from postmedial line to apex; fringe ash black with occasional pale-gray bars; underside dark gray, whitish-gray towards posterior margin, subcostal basal whitish-gray patch on some specimens, costa ash black, four or five small dirty-white spots from postmedial line to apex; orbicular and reniform spots more or less prominent dark gray. Hind wing dirty white, contrasting with forewing, numerous gray scales, darker in costal and terminal areas, terminal line contrasting dark gray, fringe pale gray; underside pale gray contrasting with forewing, discal spot dark gray, numerous gray scales, darker in dorsal and terminal areas, terminal line contrasting dark gray, fringe pale gray. *Abdomen*: Dorsum dark gray, smoothly scaled; underside dark gray, numerous white-tipped scales, smoothly scaled. *Genitalia* (Fig. 28): Apex of each arm of tegumen produced to tab-shaped lobe, juncture of two arms Y-shaped; uncus straight, drawn out to long point, long hairs sparse, denser basally; juxta widened and rounded anteriorly, laterally thickened, center U-shaped, posteriorly narrowed, drawn out to an obscure terminus; vinculum broadly V-shaped, short; valve divided, saccular region wider for basal one third, dorsal margin bent so apical two thirds narrower, heavier sclerotization at bend, narrowed part slightly curved dorsad, margins parallel, apex rounded, narrowed region densely hairy mesially; cucullar region more than two times length of saccular region, densely hairy mesially, distinctly widened to one-half length, curved dorsad, slightly narrowing towards apex, apex broadly rounded. Aedeagus (Fig. 29) straight; vesica at 90° angle, base wide, narrowing apically, forming loop 270°–360°, apically bent 90°, minute denticules at base, median diverticulum small, thin, plate-like. **Adult female** (Fig. 12): Similar to male; forewing: length 10.0–14.0 mm, mean 13.0 mm, n = 20. *Genitalia* (Fig. 40): Papilla analis lightly sclerotized, apex rounded, setae stout, numerous, conspicuous; posterior apophysis conspicuously widened near base, extending anteriorly to just beyond posterior margin of eighth segment; anterior apophysis equal length, not widened; ostium bursae funnel shaped, trapezoid-shaped plate lightly sclerotized; ductus bursae membranous, gradually narrowing to middle, widening to juncture with corpus bursae, light rugose sclerotization near juncture with corpus bursae; corpus bursae globular, membranous, scattered denticules, narrowed at insertion of ductus bursae; signum, concave, densely covered with elongate denticules.

**Figures 36–43.**
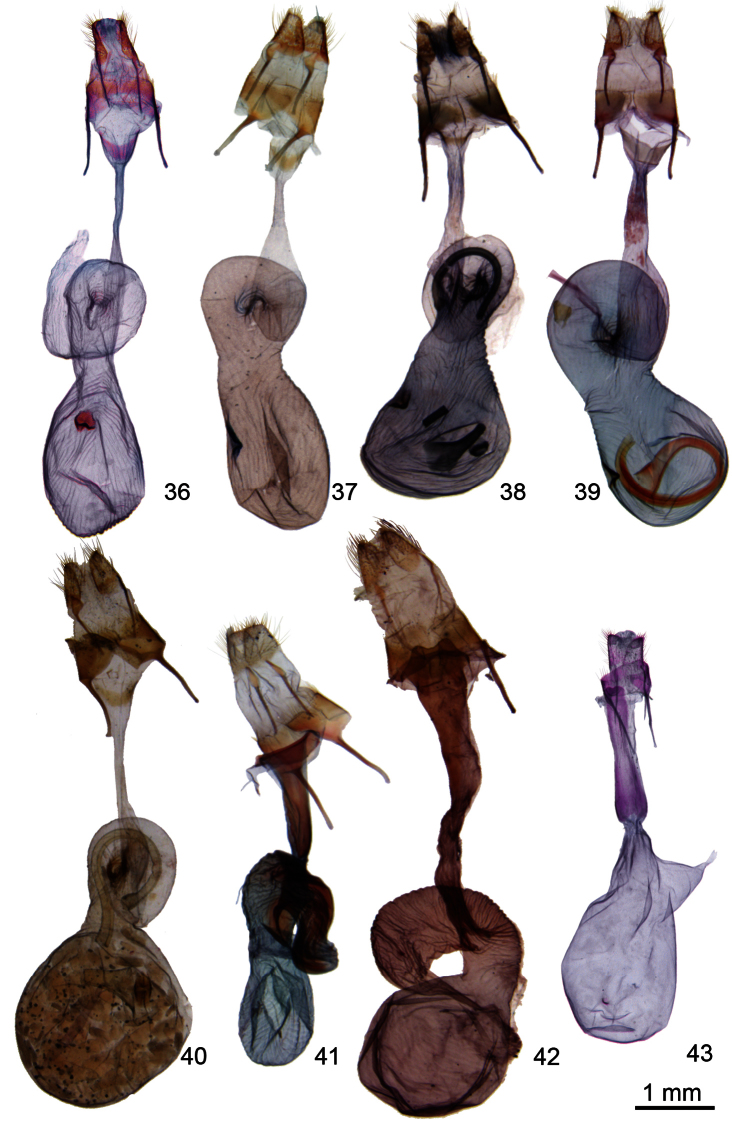
*Ogdoconta* female genitalia. **36** *Ogdoconta cinereola* **37** *Ogdoconta moreno* **38** *Ogdoconta sexta* **39** *Ogdoconta altura* **40 ***Ogdoconta satana* **41** *Ogdoconta tacna* **42** *Ogdoconta rufipenna* **43** *Ogdoconta fergusoni*.

##### Remarks.

This species is placed in the genus *Ogdoconta* on the basis of the structure of the male and female genitalia. The postmedial line is more complete on some specimens. The loop in the vesica of most specimens examined (n=8) was 270°; however the loop in one specimen was nearly 360°. The type locality was selected because it will be protected by the U.S. National Park Service into perpetuity. Metzler, Knudson, and Poole are the sole authors of this species.

##### Distribution and biology.

**Figure 44.**
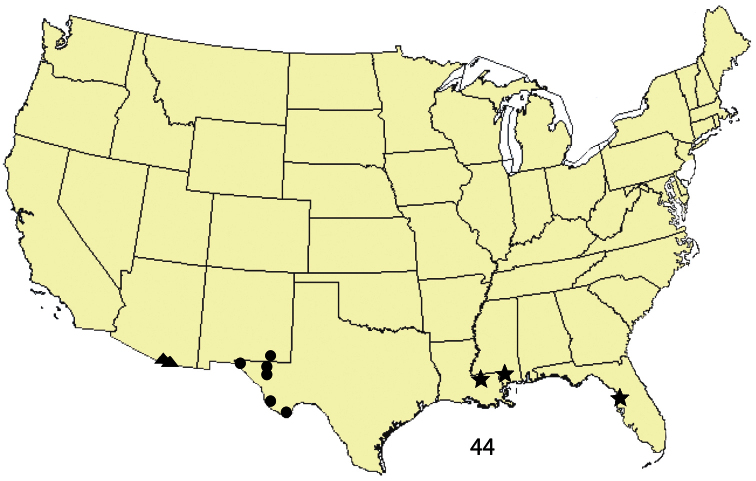
Distribution of new species of *Ogdoconta*: ● = *Ogdoconta satana*. ▲= *Ogdoconta rufipenna*. ★= *Ogdoconta fergusoni*.

This species is known from western Texas and Carlsbad Caverns National Park, Eddy County, New Mexico (Fig. 44). Its distribution into Mexico is not known. The larva and its food plant(s) are unknown. *Ogdoconta satana* is common in Carlsbad Caverns National Park, otherwise it is infrequently collected.

### *tacna* species group

#### Ogdoconta tacna

(Barnes, 1904)

http://species-id.net/wiki/Ogdoconta_tacna

Figs 14, 15 , 32, 33 , 41

Caradrina tacna Barnes 1904a: 167.

##### Type material.

*Caradrina tacna* is based on an unspecified number of syntypes. The female syntype in the USNM bearing the locality “Kerrville, Texas” is labeled and hereby designated as **Lectotype** to ensure the stability of the name.

##### Diagnosis.

The forewing of *Ogdoconta tacna* is gray brown with a slight greenish tint. The species is separable from all other species of *Ogdoconta* in North America by a pattern of fine white lines and a light scattering of white scales over a gray-brown forewing. In particular, the orbicular and reniform spots are clearly outlined by fine, dirty-white lines. Postmedial line is mostly straight and oblique from the costa to the posterior margin, although there is a slight outward pointing angulation near the bottom of the reniform spot. Postmedial line is accented with vague dark gray-green rectangles on its inner side. Subterminal area is slightly lighter than the terminal area, and the subterminal line is irregular and dull white. Terminal line consists of a series of dark rectangles accented on their inner sides by white lines. Hind wing of the male is dirty white with dark scales along the fringe and a dusting of dark scales along the costal margin. Female hind wing is more generally suffused with dark scales. White still shows through, particularly basally and along the posterior margin. Forewing length: 11.0–13.5 mm.

The male genitalia are distinctive. The cucullar part of the valve is triangular, not ovate, and there is a series of small knobs along the outer margin. Aedeagus is long, narrow, and slightly sinuous. Vesica is narrow with a tight basal loop followed by a straight region containing a double row of short, stubby spines, not found in the other North American species of *Ogdoconta*. The distal end of the vesica is another tight loop. Female genitalia are distinctive. Ostium is strongly sclerotized and the sclerotization extends the entire length of the ductus bursae. Appendix bursae contains a series of sclerotized rugosities. The distal end of the appendix bursae is not distinguishable from the beginning of the ductus seminalis. The caudal end of the corpus bursae contains the same sclerotized rugosities found in the appendix bursae.

##### Distribution and biology.

In the US, this species is only known from central and southeastern Texas. The distribution of *Ogdoconta tacna* in Mexico is not known. The larva and its food plants are unknown. Adults were collected in April and May and again in September and October. *Ogdoconta tacna* is infrequently collected.

##### Remarks.

The Lectotype designated here is the specimen illustrated as type female on plate IX, fig. 15 in Barnes and McDunnough (1912).

The shape of the valve and the knob-like projections on the valve can be seen by brushing a few scales from the protruding genitalia of male specimens.

The CNC specimen from Florida reported as *Ogdoconta tacna* (Kimball 1965, Heppner 2003) is *Ogdoconta fergusoni* Metzler & Lafontaine, new species. The specimen from Cassadaga, FL, attributed to Stanley V. Fuller, could not be located amongst Fuller’s specimens, now deposited at the McGuire Center for Lepidoptera and Biodiversity, University of Florida, and it could not be located at the Museum of Comparative Zoology, Harvard University, which is where Kimball deposited his specimens. If the Fuller specimen was examined by Kimball it is easy to speculate that the Fuller specimen is *Ogdoconta fergusoni*. The specimens identified as *“Ogdoconta* species near *tacna*” (Kons and Borth 2006) from six localities in northern and northeast Florida were not available for this study.

#### Ogdoconta fergusoni

Metzler & Lafontaine sp. n.

urn:lsid:zoobank.org:act:26626E74-FA32-48C5-A502-B91402562695

http://species-id.net/wiki/Ogdoconta_fergusoni

Figs 18, 19 , 30, 31 , 43 , 44

##### Type material.

**Holotype**: Adult male, pinned: USA, FLORIDA: Hernando County, Brookesville, N28.583, W82.333, 6-V-2002 J. Vargo. Male genitalia on slide 136355 (USNM). **Paratypes**: 3 females: MISS.,Wilkinson Co., Clark Creek Nat[ural]. Area, 31°04'22"N, 91°31'05"W, 12 July 1997, R.L.Brown. Aug-1-1981 Weyanoke, West Feliciana Par. Louisiana USA Coll. V.A. BROU. Florida 14 7[?= 14 July]. Ex Coll. Wolley-Dod. (CNC, MEM, VAB).

##### Etymology.

The scientific name *fergusoni* recognizes the contributions of Douglas Campbell Ferguson to the study of Lepidoptera and his role as a friend and mentor to many people who study Lepidoptera. The name is in the genitive case.

##### Diagnosis.

*Ogdoconta fergusoni* is the smallest North American species of *Ogdoconta*. At first glance it might be overlooked as a species of *Elaphria* Hübner, 1818, especially *Elaphria fuscimacula* (Grote, 1881) or *Elaphria grata* Hübner, 1818. Both species of *Elaphria* are reddish brown and *Ogdoconta fergusoni* is shades of gray with hints of brown and has fuscous hind wings. Historically (Kimball 1965, Heppner 2003) *Ogdoconta fergusoni* was confused with *Ogdoconta tacna*. *Ogdoconta fergusoni* is dark gray or dark reddish brown like *Ogdoconta tacna*. When *Ogdoconta fergusoni* is compared to *Ogdoconta tacna*, the forewing of *Ogdoconta tacna* is not noticeably paler in the postmedial area, *Ogdoconta tacna* is noticeably larger, the maculation of *Ogdoconta tacna* is more prominently outlined in white, and the base of the hind wings of females of *Ogdoconta tacna* have at least a few dirty-white scales. The postmedial area of the forewing of *Ogdoconta fergusoni* is noticeably paler than the postmedial area of the forewing of *Ogdoconta tacna*, *Ogdoconta fergusoni* is smaller than *Ogdoconta tacna*, and the hind wings of *Ogdoconta fergusoni* are solidly fuscous. When held obliquely to the light, the forewings of *Ogdoconta fergusoni* appear shiny.

##### Description.

**Adult male** (Fig. 18). *Head***:** Front smooth, scales white tipped gray brown; vertex, scales spatulate, 45° erect posteriorly, white tipped gray brown. Labial palpus porrect, reaching top of head; first segment dorsal scales, long, narrow, erect, pale yellow, mesial surface smooth, dirty white, ventral surface scales erect, shaggy tuft, dirty white, lateral surface smooth, scales, gray brown and white; second segment dorsal scales, dark reddish-brown, scattered white scales, smooth, mesial scales dirty white with brown and brown with white tips, ventral surface very slight tuft, scales brown with white tips; third segment scales smooth, gray brown, scattered dirty white, apex dirty white, scales smooth, gray brown, scattered dirty-white scales, apex pointed, dirty white. Haustellum coiled between labial palpi. Antenna filiform, dorsal surface scales alternating dirty white and gray brown, ventral surface naked, sensory setae sparse, setal length = 4/5 segment width. *Thorax*: Scales spatulate, white tipped gray brown, partially erect, disc smooth; underside dirty white, scales rounded, appressed, scattered long hair-like scales. Legs: fore leg dorsal surface brown, scattered dirty-white scales, ventral surface dirty white, scattered gray-brown scales, segments tipped with dirty white, tarsomeres dorsal surface brown, ventral scales dirty white, each tarsomere tipped with dirty white, mid-leg similar, tarsomeres ventral surface dirty white; hind leg dirty white, scattered gray-brown and pale-fuscous scales. Forewing: length 9.0 mm, n = 1; dorsal surface ground color gray brown with dirty-white-tipped scales, hoary. Basal line on costal half, fine, scalloped, white, incomplete; antemedial line fine, vaguely double, three elements, basal and outer elements brown, defined by white center element, scalloped; medial shade, gray brown, scales without white tips, barely darker; postmedial line not reaching costa, five elements, basal and outer elements vague, frosted, center element mostly white, lateral elements gray brown without white tips, veins brown towards costa; postmedial region contrastingly pale, pale-brown scales without white tips; subterminal line fine, waved, white; terminal line fine, white, straight, interrupted on veins; orbicular spot round, fine white outline, inconspicuous; reniform spot figure 8 shape, fine white outline, inner edge of dorsal loop extended basally to a point, dorsal loop, except basal extension, filled with light-brown scales; costa outer half marked with five white spots; fringe gray brown, white scales mark ends of veins, inconspicuous; underside pale fuscous, costal region scales mixed dirty white and gray brown, scales on veins dark, postmedial line pale gray, broadly excurved, terminal line finely dark brown; gray gradually darkening outwardly, white scales mark ends of veins. Hind wing scales fuscous-tipped pale gray, terminal line finely marked dark brown; fringe inner half dirty-white, outer half fuscous; underside base and posterior margin dirty white, gradually darkening to gray brown towards costa and outer margin; terminal line finely marked dark brown; fringe inner half dirty white, outer half fuscous. *Abdomen*: Dorsal surface pale fuscous, scales appressed, spatulate and hair-like scales mixed; basal tufts on segments one, two, and three, with gray-brown dirty-white-tipped scales, partially erect; ventral surface scales appressed, scales mixed dirty white, pale fuscous, and scattered gray brown. *Genitalia* (Fig. 30): Tegumen, each side straight, narrow, rounded at apex at junction, not modified; uncus straight, setose, slightly shorter than subscaphium, apex not narrowed, bluntly rounded; scaphium lightly sclerotized, fin-like; subscaphium sclerotized, tube-like, sub-apical scobinate patch; juxta narrow, rounded anteriorly, sub-lateral ridges strongly sclerotized, laterally indistinct, posteriorly drawn out to a long indistinct point; vinculum arms broad, broadly U-shaped, length moderate, stout; valve deeply divided, saccular region mesial surface densely hairy, gradually widened from base to one third, basal one third costa thickened, dorsal margin abruptly bent at one third, saccular area abruptly narrowed at one third, distal two thirds slightly directed ventrally, gradually widening and narrowing to bluntly rounded apex; cucullar region, one-fourth longer than saccular region, gradually widening to three-fourths length, apical one-fourth, ventral margin angled dorsally to broadly rounded apex, mesial surface of apical one-half densely hairy, ventral margin at three-fourths drawn out to a prominent finger-like projection. Aedeagus (Fig. 31) stout, straight. Vesica basally wide, broadly curved 180°, outer margin of loop lightly sclerotized with minute scobinations, narrowed apically, basal sclerite, very small, linear, prominent denticular teeth in double row, broad diverticulum before top of loop. **Adult female** (Fig. 19). Similar to male except wing dorsal surface more reddish; forewing: length 9.0–10.5 mm, mean 9.5 mm, n = 3. *Genitalia* (Fig. 43): Papilla analis lightly sclerotized, apex rounded, setae gradually denser towards apex; posterior apophysis extending anteriorly to just beyond posterior margin of eighth segment; eighth segment sclerotized ring, anterior margin extended into base of anterior apophyses; anterior apophysis length = 2× posterior apophysis; lamella postvaginalis sclerotized, excurved caudally, shallow concavity mesially, posterior margin with numerous short stiff hair-like tiny projections; surface densely covered with pointed spicules, density of spicules and length of points decreases anteriorly; ductus bursae sclerotized entire length, sclerotization weaker at midpoint, anterior half gradually widening, abruptly narrowing to short membranous section at junction with corpus bursae; corpus bursae posterior end with wide weakly sclerotized rugosities, bulbous, lengthened anteriorly; signum concave, approximately round, covered with pointed spicules, surrounded by bluntly-rounded cobble-like spicules, prominence of rounded spicules decreases as distance from signum increases; appendix bursae at posterior end of corpus bursae, directed laterally, more or less distinct, funnel-shaped, not coiled, narrowed to distinct ductus seminalis.

##### Remarks.

This new species is placed in the genus *Ogdoconta* on the basis of the shape of the male and female genitalia, and the appearance of the adult moth. The medial shade, visible under magnification, is detected by the absence of dirty-white-tipped scales. The female specimen in the CNC from Florida (Wolley-Dod) has a darker postmedial area than the other specimens in the type series; however the genitalia of this specimen leave no doubt that it is *Ogdoconta fergusoni*.

The hair-like projections on the posterior margin of the lamella postvaginalis and the pointed ends of the spicules are very small. They can be seen with a compound microscope at 60 × or greater magnification. The number of hair-like projections and the number of pointed ends which are visible increases as magnification increases.

This species was misidentified as *Ogdoconta tacna* (Kimball 1965, Heppner 2003). Metzler & Lafontaine are the sole authors of this species.

##### Distribution and biology.

This species is recorded from Florida, southern Mississippi, and southern Louisiana (Fig. 44). The larva and its food plants are unknown. *Ogdoconta fergusoni* is infrequently collected.

### *rufipenna* species group

#### Ogdoconta rufipenna

Metzler, Knudson, & Poole sp. n.

urn:lsid:zoobank.org:act:C0C5F702-F47C-4078-B995-A2B116A3F4D3

http://species-id.net/wiki/Ogdoconta_rufipenna

Figs 16, 17 , 34, 35 , 42 , 44

Ogdoconta sp. not *Ogdoconta lilacina* (Druce, 1890), (Lafontaine and Schmidt 2010): 66.

##### Type material.

**Holotype**: Adult male, pinned: Madera Canyon 5800’, Santa Rita Mts., Santa Cruz Co., Ariz., 6 July 1960, J. G. Franclemont. (CUIC). **Paratypes**: 121 males and 50 females: AZ Santa Cruz Co., Sycamore Canyon, 4000’, 6 August, 1991, UV light., C. D. Ferris leg. Madera Canyon 4880’, Santa Rita Mts., Santa Cruz Co., Ariz., 26 June 1960–24 July 1960, J. G. Franclemont. Madera Canyon 5800’, Santa Rita Mts., Santa Cruz Co., Ariz., 27 June 1960–21 July 1960, J. G. Franclemont. Ariz., Santa Cruz Co., Madera Canyon, Santa Rita Mts., 5100’, July 10–26, 1964, D.R. Davis. Ariz., Washington Mts., B.P. Clark Donor. USA:AZ: Cochise Co. Huachuca Mts. 5354 Ash Cyn. Rd., 0.5miW Hwy 92 2.IX.1991 5100’ N.McFarland UV light. USA:AZ: Cochise Co. Huachuca Mts. 5354 Ash Cyn. Rd., 0.5miW Hwy 92 20.VII.1988 5100’ N.McFarland UV light. Sierra Vista, ARIZ 9-IV-1967 R. F. Sternitzky. Sierra Vista, ARIZ 13.IX.1967 R. F. Sternitzky. Miller Canyon Huachuca Mts., Arizona 23.V.66 R. F. Sternitzky. Miller Canyon Huachuca Mts., Arizona 30.VII.67 R. F. Sternitzky. July 6, 1958, Sunnyside, W. side, Huachuca Mt’s., Cochise Co., Arizona Lloyd M. Martin, 15 watt black light. July 7, 1958, Sunnyside, W. side, Huachuca Mt’s., Cochise Co., Arizona Lloyd M. Martin, 15 watt black light. July 8, 1958, Sunnyside, W. side, Huachuca Mt’s., Cochise Co., Arizona Lloyd M. Martin, 15 watt black light. July 9, 1958, Sunnyside, W. side, Huachuca Mt’s., Cochise Co., Arizona Lloyd M. Martin, 15 watt black light. July 11, 1958, Sunnyside, W. side, Huachuca Mt’s., Cochise Co., Arizona Lloyd M. Martin, 15 watt black light. July 13, 1958, Sunnyside, W. side, Huachuca Mt’s., Cochise Co., Arizona Lloyd M. Martin, 15 watt black light. July 14, 1958, Sunnyside, W. side, Huachuca Mt’s., Cochise Co., Arizona Lloyd M. Martin, 15 watt black light. July 16, 1958, Sunnyside, W. side, Huachuca Mt’s., Cochise Co., Arizona Lloyd M. Martin, 15 watt black light. July 17, 1958, Sunnyside, W. side, Huachuca Mt’s., Cochise Co., Arizona Lloyd M. Martin, 15 watt black light. July 18, 1958, Sunnyside, W. side, Huachuca Mt’s., Cochise Co., Arizona Lloyd M. Martin, 15 watt black light. Ramsey Canyon Huachuca Mts., 28.VIII.66 R. F. Sternitzky. Ramsey Canyon Huachuca Mts., 15.IX.67 R. F. Sternitzky. Sept. 6, 1959, Ramsey Canyon, Huachuca Mt’s., Cochise Co., Ariz., 15 watt black light, Lloyd M. Martin. July 7–17, 1958, Sunnyside, W. side, Huachuca Mt’s., Cochise Co., Arizona Tom W. Davies, 15 watt black light. AZ, Cochise Co, 6050 ft., Huachuca Mts., Copper Canyon, UV/MV lights, Oak woodland habitat, 24 July 2010, B. Walsh leg. Madera Canyon 4880’, Santa Rita Mts., Santa Cruz Co., Ariz., 19 June 1960, 25 June 1960, 26 June 1960, 27 June 1960, 1 July 1960, 3 July 1960, 4 July 1960, 5 July 1960, 6 July 1960, 10 July 1960, 20 July 1960, 22 July 1960, 23 July 1960, 24 July 1960, 27 July 1960, 1 August 1960, J. G. Franclemont. Madera Canyon 4880’, Santa Rita Mts., Santa Cruz Co., Ariz., 26 June 1959, 1 July 1959, 4 July 1959, 5 July 1959, 7 July 1959, 9 July 1959, 11 July 1959, 12 July 1959, 13 July 1959, 14 July 1959, 16 July 1959, 18 July 1959, 19 July 1959, 20 July 1959, 22 July 1959, 23 July 1959, 24 July 1959, 27 July 1959, 13 August 1959, 15 August 1959, 24 August 1959, 25 August 1959, J. G. Franclemont. Madera Canyon 5800’, Santa Rita Mts., Santa Cruz Co., Ariz., 21 June 1960, 26 June 1960, 27 June 1960, 28 June 1960, 1 July 1960, 5 July 1960, 7 July 1960, 8 July 1960, 13 July 1960, 21 July 1960, 22 July 1960, 26 July 1960, 29 July 1960, J. G. Franclemont. ARIZ., Cochise Co., Huachuca Mts. 6550’, Carr Canyon Rd. 4.8 mi from jct Ariz. 92, 15 August 1966, at U. V. light, Robert G. Beard. (USNM, CDF, CNC, CUIC, EHM, LACM, JBW).

##### Etymology.

The scientific name *rufipenna* comes from the Latin rufus = reddish and the Latin penna = wing. The name *rufipenna*, red wing, refers to the burgundy (= reddish-brown) color of the adult forewings. It is used as a singular adjective.

##### Diagnosis.

*Ogdoconta rufipenna* is a large North American species of *Ogdoconta*. *Ogdoconta rufipenna* is easily identified by a uniform burgundy-colored forewing with faint fine white lines. Forewing has few distinct markings, although all marks and lines (except the claviform spot) are present as fine white lines. No other North American species of *Ogdoconta* shares these color characteristics. Valve is distinguished by a large thumb-like projection from the lower angle of the apex of the cucul- lar region of the valve. Cucullar part of the valve is narrower than the other North American species of *Ogdoconta*, and the cucullar part is neither triangular nor ovate. Aedeagus is thin, long, and slightly sinuous, like *Ogdoconta tacna*. Vesica has a tight basal loop, similar to *Ogdoconta tacna*, but the vesica of *Ogdoconta rufipenna* has no spines, whereas the vesica of *Ogdoconta tacna* has two rows of spines between the first and second loops. Vesica of *Ogdoconta rufipenna* has a prominent curved diverticulum; the vesica of *Ogdoconta tacna* does not have a diverticulum. The distal end of the vesica of *Ogdoconta rufipenna* forms a wide loop. Lamella postvaginalis and the caudal half of the ductus bursae of *Ogdoconta rufipenna* are strongly sclerotized; there are no sclerotized rugosities in the appendix bursae. In *Ogdoconta tacna* the lamella postvaginalis is sclerotized, and there are strongly sclerotized rugosities in the appendix bursae. In the other North American species of *Ogdoconta*, the lamella postvaginalis is not strongly sclerotized.

##### Description.

**Adult male** (Fig. 16). *Head*: scales burgundy, spatulate white tipped, vertex scales erect, front scales smooth. Labial palpus dark reddish-brown, scattered white scales, erect, basal segment with ventral tuft, second segment smooth scaled, ventral mesial margin white, apical segment smooth scaled, white tipped. Haustellum coiled between labial palpi. Antenna filiform, sensory setae inconspicuous, dorsally each segment alternating pale and dark, smooth scaled, ventrally brown. *Thorax*: burgundy, spatulate white-tipped scales; underside dirty white, scales appressed, scattered long hair-like scales. Legs dorsally dark reddish-brown, scattered white scales, ventrally admixture of dirty-white and dark reddish-brown, smoothly scaled, sparse long hair-like whitish scales; tarsomeres white tipped. Forewing: length 10.5–16.0 mm, mean 14.5 mm, n = 117; dorsal surface ground color uniformly burgundy, scattered white scales, hoary. Basal line fine, inconspicuous at costa, dorsally absent, white; antemedial line fine, white from costa to below cell, dorsally absent, sometimes fused to orbicular spot; medial shade absent; postmedial line fine, white from costa to below cell; subterminal line scalloped, obscure, marked with white and dark scales; terminal line fine, white, scalloped; orbicular spot round, finely outlined with white, filled with burgundy; reniform spot kidney shaped, elongated dorsally towards orbicular spot, finely outlined with white, filled with a vague vertical white line and burgundy; costa with five small white spots from antemedial line to postmedial line; fringe dark reddish brown, white tipped; underside dark reddish brown, costa and terminal areas hoary with white scales, subterminal line white, marking edge of terminal area; terminal line dark; fringe basal line pale, two-toned dark reddish brown, white tipped. Hind wing dark reddish brown, hoary, veins lined with dark, terminal line dark, fringe two-toned, basally umber, distally paler, white tipped; underside umber, hoary, subterminal line dark, terminal line dark, fringe basal line pale, umber, white tipped. *Abdomen*: Dorsum with two basal tufts burgundy, scales white tipped, elsewhere dark reddish brown, hoary, smoothly scaled, valves (if protruding) dark yellow; underside dark reddish brown, hoary, scales appressed. *Genitalia* (Fig. 34): Tegumen, each side rounded at apex, not modified; uncus straight, setose, scarcely shorter than subscaphium, apex slightly down turned, pointed; scaphium basally sclerotized, laterally membranous, subscaphium sclerotization increasing distally, tube-like; juxta rounded anteriorly, laterally indistinct, posteriorly drawn out to a long slightly thickened point, with or without a thin keel; vinculum V-shaped, length moderate, stout; valve deeply divided, saccular region gradually widened from base to one half, dorsal margin abruptly bent at one half, saccular area abruptly narrowed at one half, gradually widening and narrowing to bluntly rounded apex, minute denticules on dorsal half at bend, dense, narrowed apical region densely hairy mesially; cucullar region one fifth longer than saccular region, subtly curved, costal and ventral margins parallel almost to apex, apex swollen, capitate, rounded, densely hairy mesially, large thumb-like projection from the lower angle of apex. Aedeagus (Fig. 35) long, narrow, slightly sinuous, last one fourth densely covered with minute denticules; vesica, gradually narrowing, first loop tight, second loop elongate, median diverticulum elongate, curved, with minute denticules, dense, inside first curve of loop, minute denticules, dense, outside curve of second loop. **Adult female** (Fig. 17). -Similar to male. Forewing: length 10.5–15.5 mm, mean 14.5 mm, n = 47; *Genitalia* (Fig. 42): Papilla analis lightly sclerotized, apex rounded, setae gradually denser towards apex; posterior apophysis extending anteriorly to just beyond posterior margin of eighth segment; anterior apophysis slightly stouter than posterior apophysis but equal in length; lamella postvaginalis rounded, posterior margin slightly concave, strongly sclerotized, covered with minute denticules; ostium bursae strongly sclerotized, inside of opening with numerous minute denticules, narrowing anteriorly; ductus bursae with caudal half sclerotized on ventral side, cephalad half membranous, narrowed anteriorly, inconspicuous S-shaped bend, lightly sclerotized to left; appendix bursae attached where ductus bursae meets corpus bursae, bulbous, not coiled, membranous, surface wrinkled, infrequent indistinct patches of minute denticules; ductus seminalis distinct, directed anteriorly; corpus bursae bulbous, elongate, membranous, surface wrinkled, and patches of minute denticules, scattered, obscure; signa, if present, obscure.

##### Remarks.

This new species is placed in the genus *Ogdoconta* on the basis of the shape of the male and female genitalia. The large thumb-like projection on the valve of the male can be seen by brushing a few scales from the protruding genitalia of male specimens. This species was misidentified as *Ogdoconta lilacina* on a web site detailing the moths of southeastern Arizona(Walsh 2011). Metzler, Knudson, & Poole are the sole authors of this species.

##### Distribution and biology.

This species is recorded from Santa Cruz and Cochise Counties in southeastern Arizona (Fig. 44). Its distribution in Mexico is not known. The larva and its food plants are unknown. *Ogdoconta rufipenna* is moderately common in specific locations in Santa Cruz and Cochise counties, Arizona.

## Discussion

In preparation of this document, we examined the types of all species of *Ogdoconta*, which were described from the New World.

The coordinates for latitude and longitude are reported exactly as they are on the specimens. These data are easily converted to any other format by using one of many conversion sites on the World Wide Web, such as CSGNetwork.com (2012).

The specimens from Carlsbad Caverns National Park were collected during a ten-year study of the moths in the Park. This is the fifth in a series of papers (Metzler et al. 2010, Metzler and Knudson 2011, Metzler et al. in press, Metzler and Forbes 2012) detailing the moths of Carlsbad Caverns National Park, and *Ogdoconta satana* is the fourth species described as a result of the study. Carlsbad Caverns National Park was selected as the type locality of *Ogdoconta satana* because the site will be protected by the National Park Service into perpetuity.

## Supplementary Material

XML Treatment for Ogdoconta

XML Treatment for Ogdoconta cinereola

XML Treatment for Ogdoconta moreno

XML Treatment for Ogdoconta sexta

XML Treatment for Ogdoconta altura

XML Treatment for Ogdoconta satana

XML Treatment for Ogdoconta tacna

XML Treatment for Ogdoconta fergusoni

XML Treatment for Ogdoconta rufipenna
